# Epidemiological characteristics and spatiotemporal analysis of hand-foot-mouth diseases from 2010 to 2019 in Zibo city, Shandong, China

**DOI:** 10.1186/s12889-021-11665-0

**Published:** 2021-09-08

**Authors:** Lili Liu, Ling Wang, Chang Qi, Yuchen Zhu, Chunyu Li, Yan Jia, Kaili She, Tingxuan Liu, Yan Zhang, Feng Cui, Xiujun Li

**Affiliations:** 1 Department of Biostatistics, School of Public Health, Cheeloo College of Medicine, Shandong University, Jinan, 250012 Shandong China; 2 Institute for Infectious Disease Control and Prevention, Zibo Center for Disease Control and Prevention, Zibo, 255026 Shandong China; 3 Zibo Center for Disease Control and Prevention, Zibo, 255026 Shandong China

**Keywords:** Hand-foot-mouth disease, Space-time clustering, Autocorrelation analysis, Spatial epidemiology, Shandong province

## Abstract

**Background:**

Hand-foot-mouth disease (HFMD) is a global public health issues, especially in China. It has threat the health of children under 5 years old. The early recognition of high-risk districts and understanding of epidemic characteristics can facilitate health sectors to prevent the occurrence of HFMD effectively.

**Methods:**

Descriptive analysis was used to summarize epidemic characteristics, and the spatial autocorrelation analysis and space-time scan analysis were utilized to explore distribution pattern of HFMD and identify hot spots with statistical significance. The result was presented in ArcMap.

**Results:**

A total of 52,095 HFMD cases were collected in Zibo city from 1 Jan 2010 to 31 Dec 2019. The annual average incidence was 129.72/100,000. The distribution of HFMD was a unimodal trend, with peak from April to September. The most susceptible age group was children under 5 years old (92.46%), and the male-to-female ratio is 1.60: 1. The main clusters were identified in Zhangdian District from 12 April 2010 to 18 September 2012. Spatial autocorrelation analysis showed that the global spatial correlation in Zibo were no statistical significance, except in 2012, 2014, 2015, 2016 and 2018. Cold spots were gathered in Boshan county and Linzi district, while hot spots only in Zhangdian District in 2018, but other years were no significance.

**Conclusion:**

Hot spots mainly concentrated in the central and surrounding city of Zibo city. We suggest that imminent public health planning and resource allocation should be focused within those areas.

## Background

Hand-foot-mouth disease (HFMD), an infectious disease caused by enterovirus, mainly include coxsackie virus 16(CoxA16) and enterovirus71 (EV-71) [1–4]. New types of virus (CV-6, CV-10) have emerged in recent years [5, 6]. Since New Zealand first reported HFMD in 1957, HFMD occurred around the world, especially in the Asia-Pacific region [7–11]. Most children recover spontaneously within a week, while a small proportion of children may cause complications such as myocarditis, pulmonary edema and aseptic meningitis [12]. Unfortunately, due to the lack of specific treatment and vaccine, some severe cases developed rapidly, eventually leading to death [13].

According to a largest population-scale HFMD study in China, the average annual incidence reached 120 per 100,000 in 2010–2012, with 350–900 deaths per year, which seriously threaten the health of Chinese children under 5 years old [14]. Chinese government listed HFMD as a notifiable Class C infectious disease in May 2010 and healthcare institutions are required to report it on the Notifiable Infectious Diseases Reporting Information System (NIDRIS) within 24 h [15]. In 2015, HFMD vaccine specifically developed for EV-A71 was sold on the Chinese market, but there was no report that the implementation of the vaccine could reduce the incidence of the HFMD [5].

Therefore, it is urgency to timely understand the epidemic characteristics and spatiotemporal distribution pattern of HFMD, allowing for disease prevention and control in advance against the key populations and regions for associated authorities [16].

Compared with traditional methods, spatiotemporal analysis not only can explore the synthesis of disease location and time, but also help public health authorities implement timely surveillance and intervention for HFMD in the right place by identifying when and where the incidence is highest. Real time space-time surveillance system would help in identifying areas and populations at high risk and then formulating and implementing appropriate regional public health intervention strategies to prevent and control the outbreaks. It has been employed to explore cluster areas of HFMD in previous studies, such as Sichuan Province, Beijing city, Shandong Province [17–20]. However, as far as we know, previous studies focused on the spatial and temporal distribution of HFMD were limited in Zibo city, and a full understanding in terms of the spatial and temporal characteristics of HFMD has not yet been established. Besides, the spatiotemporal distribution features vary in different regions [21], owing to geographical location, economic conditions, social factors and climatic conditions. Therefore, this study aims to explore spatiotemporal pattern of HFMD based on the surveillance data of Zibo city from 2010 to 2019 for providing appropriate public health measures and strategies to prevent and control HFMD.

## Methods

### Study area

**Fig. 1:**
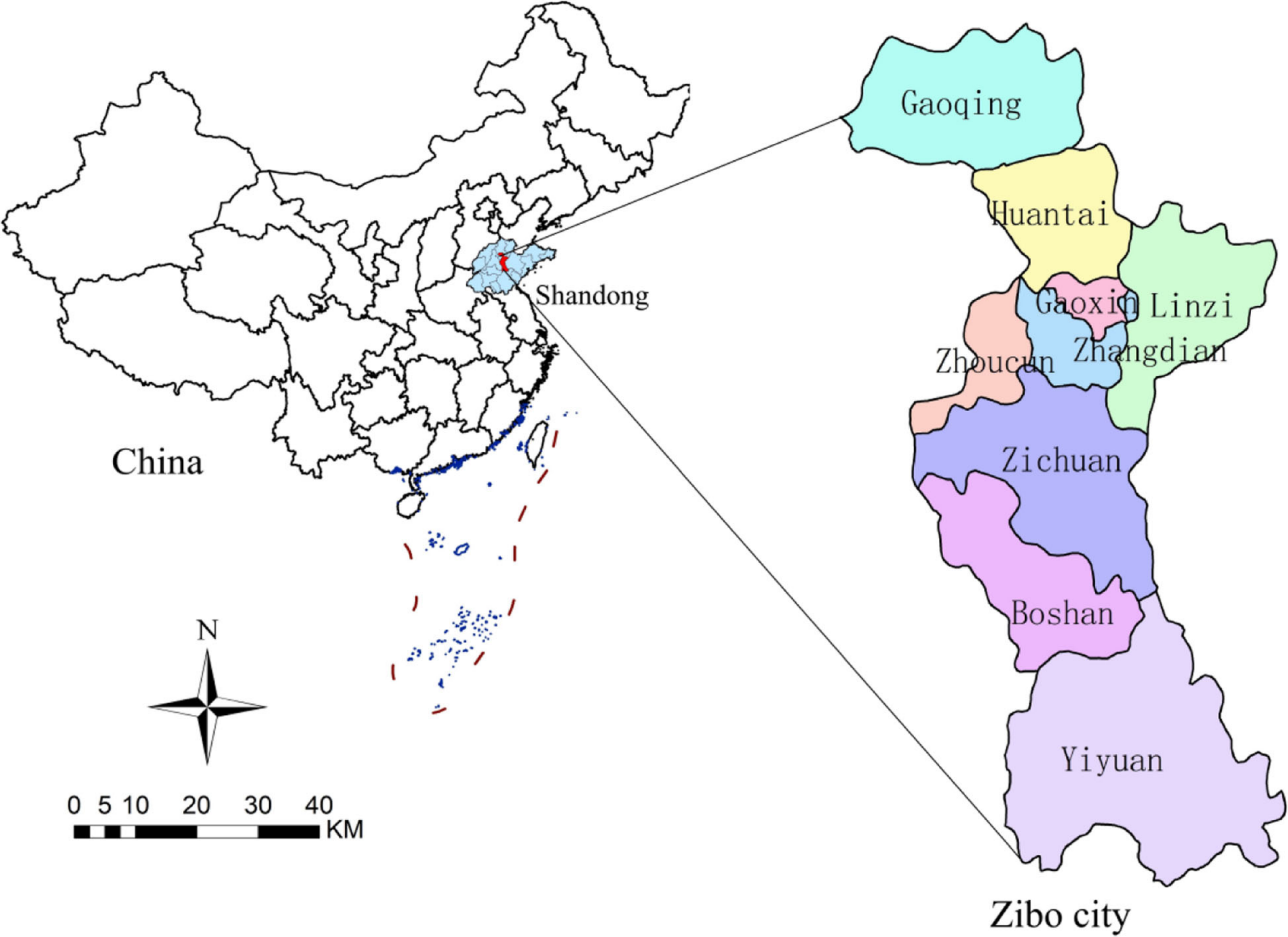
Geographical location of Zibo city, Shandong Province, China. (The author drew this map by ArcGIS10.5 software)

Zibo is a central city in the Shandong Province of China, located between latitude 35°55′ N and 37°17′ N, and longitude 117°32′ E and 118°31′ E (Fig. 1). Zibo city has a temperate monsoon climate. By 2019, Zibo city consists of five districts (Zhangdian districts, Zichuan districts, Zhoucun districts, Linzi districts and Boshan districts) and three counties (Hengtai county, Gaoqing county and Linyi county), covering an area of 5965 km^2^, with a permanent resident population of 4.702 million [22].

### Data sources and visualization

Daily reported HFMD cases from 1 Jan 2010 to 31 December 2019 were obtained from Zibo Center for Disease Control and Prevention. All HFMD cases are reported online to the infectious disease surveillance system within 24 h of diagnosis, and confirmed based on the unified diagnostic criteria issued by the Ministry of Health of China [14]. The information entered into the system includes age, sex, status and date of symptom onset [22]. The demographic data for each county of Zibo city was downloaded from Zibo Statistical Yearbook (http://tj.zibo.gov.cn/module). The incidence of HFMD is calculated as the ratio of the number of cases to the number of permanent residents during the study period. In addition, as the Gaoxin District is under the jurisdiction of Zhangdian District, cases of Gaoxin District were classified as Zhangdian District.

Excel 2016 and R 4.0.2 were used for analyzing and processing basic data, OpenGeoDa was applied for spatial autocorrelation analysis and SaTScan 9.6 was used for spatiotemporal clustering analysis. We matched administrative codes of home addresses of HFMD cases to the map codes of the counties, combined with the map of cases, and the results were shown in ArcGIS 10.5 [23].

### Statistical analysis

The HFMD incidence was defined as the number of cases divided by the total number of the population in each county, and represented by the different colors on the map combined with ArcGIS 10.5. The darker the color, the higher the incidence. The detailed group was shown in the Fig. 4.

Global spatial autocorrelation aimed at exploring whether there is correlation among the whole study region from the global perspective, which describes the spatial figure of attribute values in the whole region. It was measured by comparing the similarity of observed values in adjacent spatial positions [16].

Compared with global autocorrelation, the advantage of local spatial autocorrelation is that it can evaluate significant local spatial clustering around a single location by combining LISA map, and visualize them clearly in a form of significance and cluster maps. Local spatial autocorrelation calculates the Local Moran’s I index for each county, which indicates the degree of significant difference between the county of interest and its neighbors in the predefined neighborhood context [20].

LISA map can be divided into four types: High-High mode (HH), High-Low mode (HL), Low-High mode (LH) and Low-Low mode (LL) respectively. The HH means high incidence areas in the cluster cities are also surrounded by high incidence areas. Similarly, HL represents the aggregation area is high incidence area, and the surrounding areas were low incidence areas [24].

### Space – time scan analysis

Space – time scan methods include spatiotemporal scan and purely space scan method in this study. Both methods take county as the smallest scanning unit, using the three-dimensional dynamic changing cylinder scan window to analyze data. Purely space scan statistics imposes a circular window on the map. The spatiotemporal scan analysis sets radius of the circular window as the geographic position and the size of the region, and the height correspond to the scanning time [25].

We set 30% the total population and scanning time as maximum spatial and time scanning window respectively. Monte Carlo was used to calculate *P* value of the test statistics, and the simulation times were set to 999. Both methods were under hypothesis of Poisson distribution, we calculated the theoretical cases of inner scanning window, combined with the actual cases to construct logarithmic likelihood ratio (*LLR*) statistics which is used to evaluate abnormal incidence degree. When the hypothesis test result of *LLR* was *P* < 0.05, the difference in relative risk (*RR*) between inside and outside the scan window was statistically significant. The cluster group with maximum *LLR* as the main cluster, and others are secondary clusters in order [23]. $u(A)=\frac{m_a}{m_G}\cdotp {n}_G$

$$ LLR=\frac{L_A}{L_0}=\frac{{\frac{n_A}{u(A)}}^{n_A}{\left(\frac{n_{G-}{n}_A}{u(G)-u(A)}\right)}^{n_{G-}{n}_A}}{{\left(\frac{n_G}{u(G)}\right)}^{n_G}} $$

Where *u*(*A*), *m*_*a*_ represent the expected number of cases and population of window A under the random hypothesis respectively. *m*_*G*_ represents the total population in the study area. L_0_, L_A_ represent the likelihood function value under hypothesis and window A respectively; *n*_*G*_ represents the total number of cases in the space-time range. *n*_*A*_ *r* epresents the actual number of cases in window A, u(G) =*Σu*(*A*).

## Results

### Descriptive analysis

In 2010–2019, we collected 52,095 HFMD cases in Zibo city, included 31,771 males and 20,324 females, with a sex ratio of 1.6:1. The majority of the patients were children aged 0–5 years (91.62%). Scattered children were the dominant infected population, accounting for as much (55.31%), followed by kindergarten children (39.19%) (Table 1).

**Table 1:** Demographic characteristics of patients with HFMD in Zibo city, 2010 ~ 2019

	2010	2011	2012	2013	2014	2015	2016	2017	2018	2019	percentage (%)
Sex											
Male	5161	3228	3618	2359	4509	2492	3523	2165	2936	1780	60.99
Female	3129	1975	2148	1544	3036	1577	2363	1461	1888	1203	39.01
Age											
0–5	7782	4818	5315	3660	6726	3797	5327	3283	4385	2639	91.62
6–14	489	377	435	227	784	252	538	313	385	318	7.91
> 14	19	8	16	16	35	20	21	30	54	26	0.47
Work											
Scattered	4394	2513	2956	2409	3796	2654	3249	2220	3270	1352	55.31
Kindergarten	3617	2468	2528	1331	3205	1228	2290	1160	1222	1365	39.19
Students	264	217	271	149	514	173	328	222	289	251	5.14
Others	15	5	11	14	30	14	19	24	43	15	0.36
Severe Case											
Yes	2	5	0	0	2	0	2	26	3	2	0.08
No	8288	5198	5759	3903	7540	4069	5884	3600	4821	2981	99.90
No record	0	0	7	0	3	0	0	0	0	0	0.02
Total	8290	5203	5766	3903	7545	4069	5886	3626	4824	2983	100

**Fig. 2:**
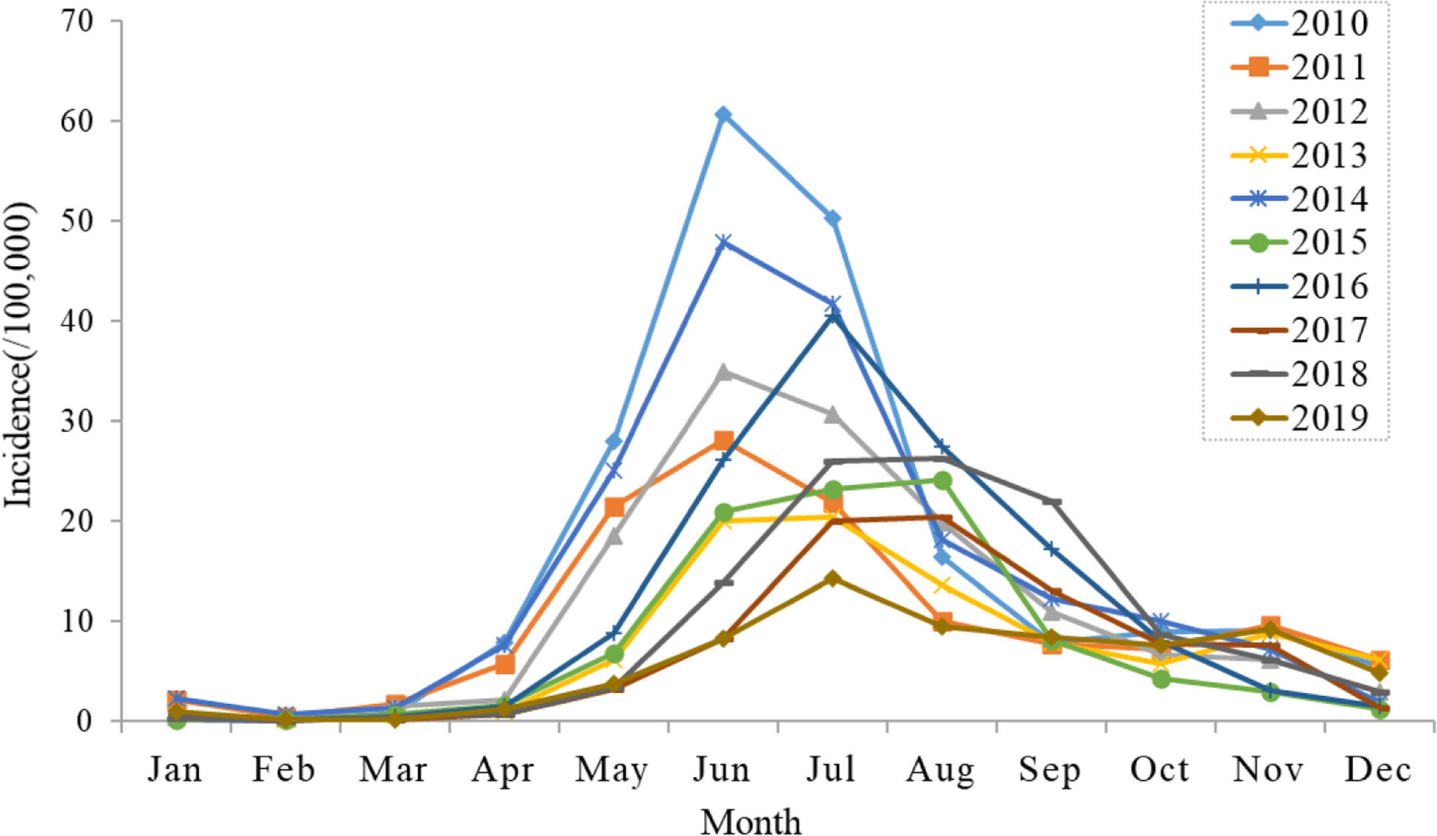
Yearly and monthly HFMD incidence in Zibo city, Shandong Province, China, 2010–2019

**Fig. 3:**
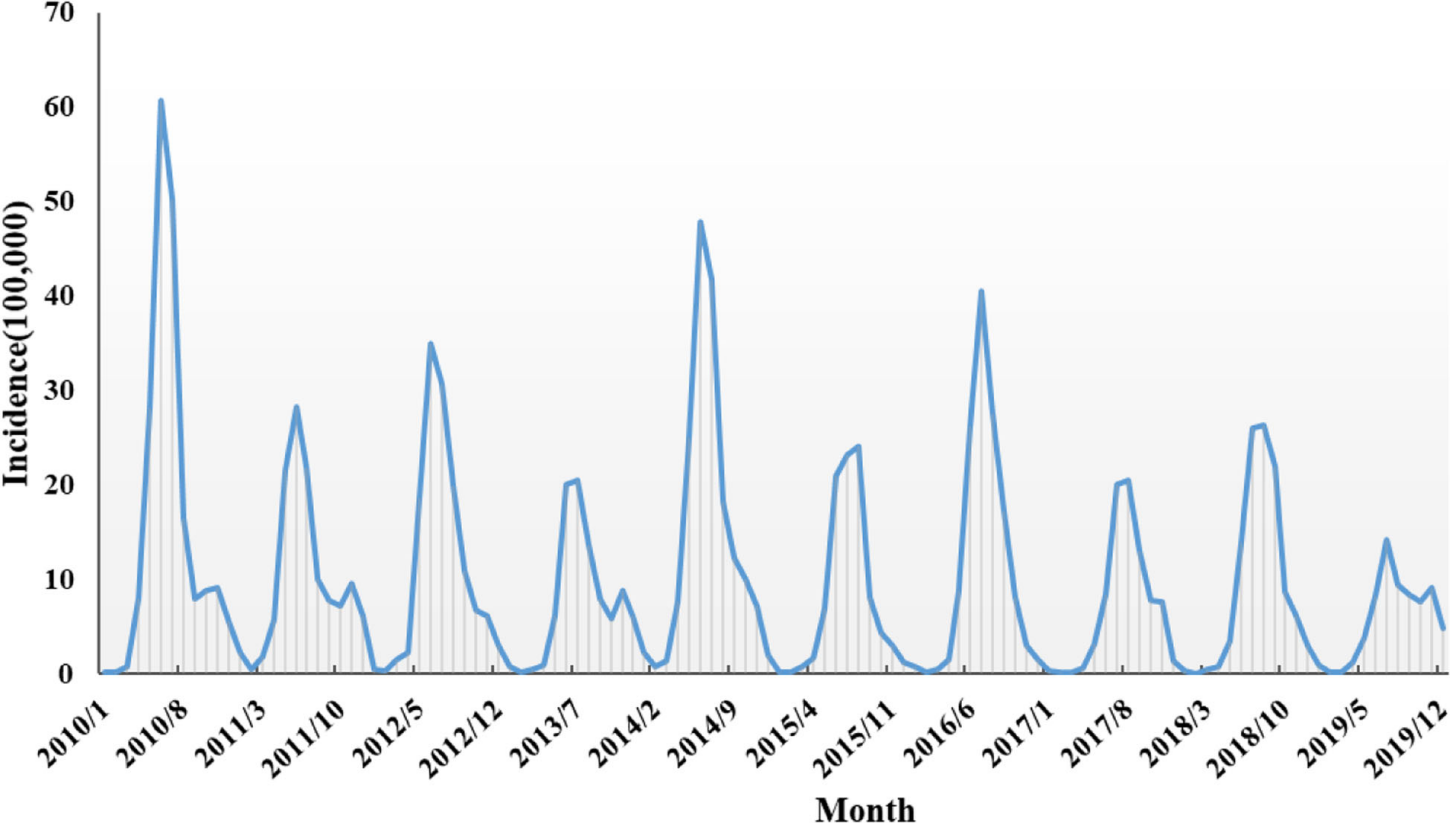
Sequence diagram of monthly HFMD cases from January 2010 to December 2019 in Zibo city. (The author drew this map by ArcGIS10.5 software)

Yearly HFMD incidence during 2010–2019 were presented in Fig. 2. The occurrence of HFMD shows seasonality, the peak appears from April to September during the study period, presenting a unimodal distribution. Additionally, Fig. 3 revealed that there is a periodicity about 2 years. The incidence interval was in a range of 7.88/100,000 (2019) to 291/100,000 (2010), with the annual average incidence 129.72/100,000.

**Fig. 4:**
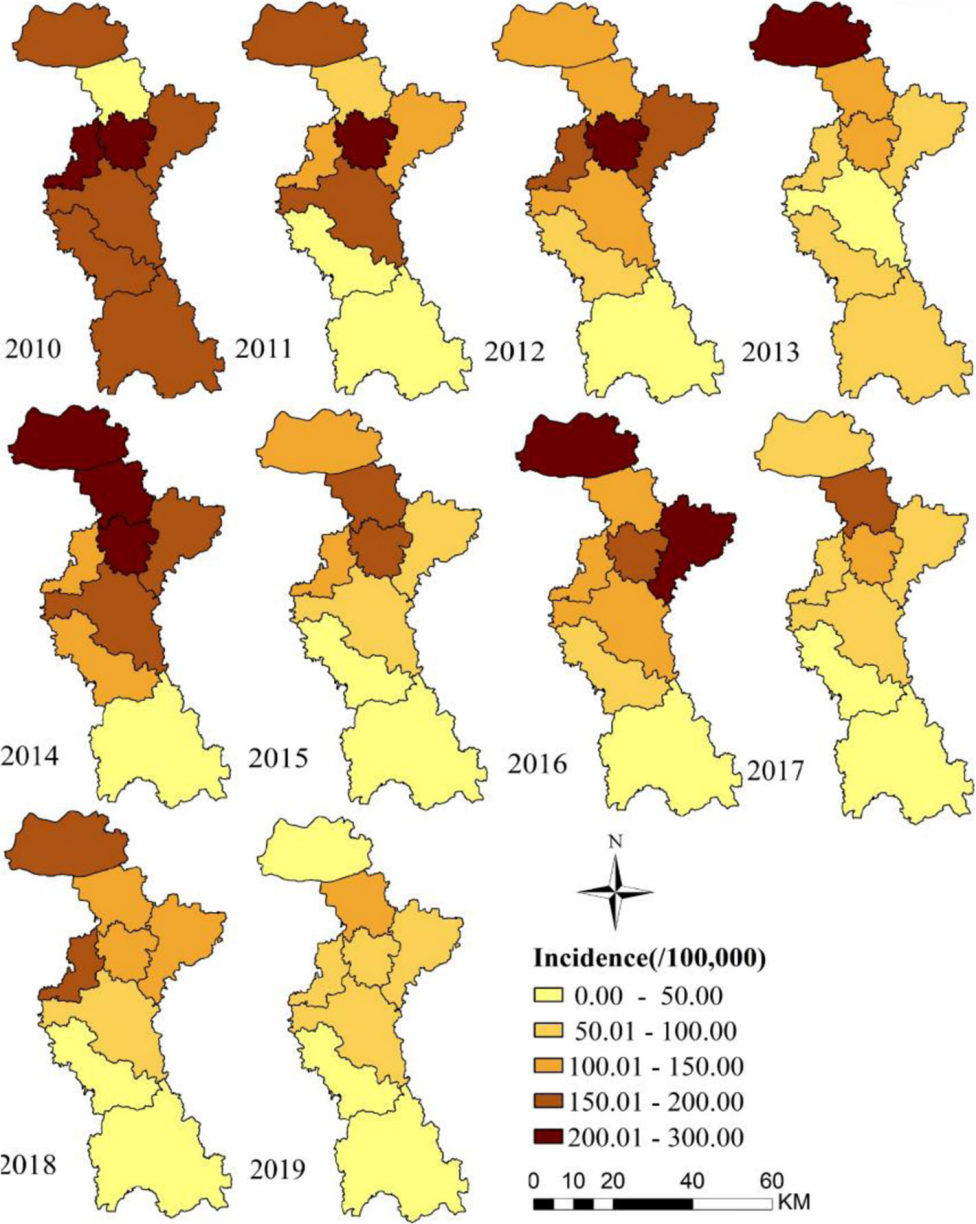
Annual incidence (/100,000) of HFMD at county level in Zibo city, Shandong Province, 2010–2019. (The author drew this map by ArcGIS10.5 software)

The spatial distribution of annual HFMD cases revealed that regions with higher incidence than average nine-year incidence are different (Fig. 4). The first 3 years mainly distributed in Zhangdian District in central Zibo city, focused on Gaoqing county in 2013, and on Gaoqing county, Hengtai county and Zhangdian District in 2014. In 2015 and 2016, with the exception of Gaoqing county, high incidence area also included Linzi District. Since 2017, the incidence presents the downward trend compared to first 7 years, focused on Hengtai county.

### Spatial autocorrelation analysis

**Fig. 5:**
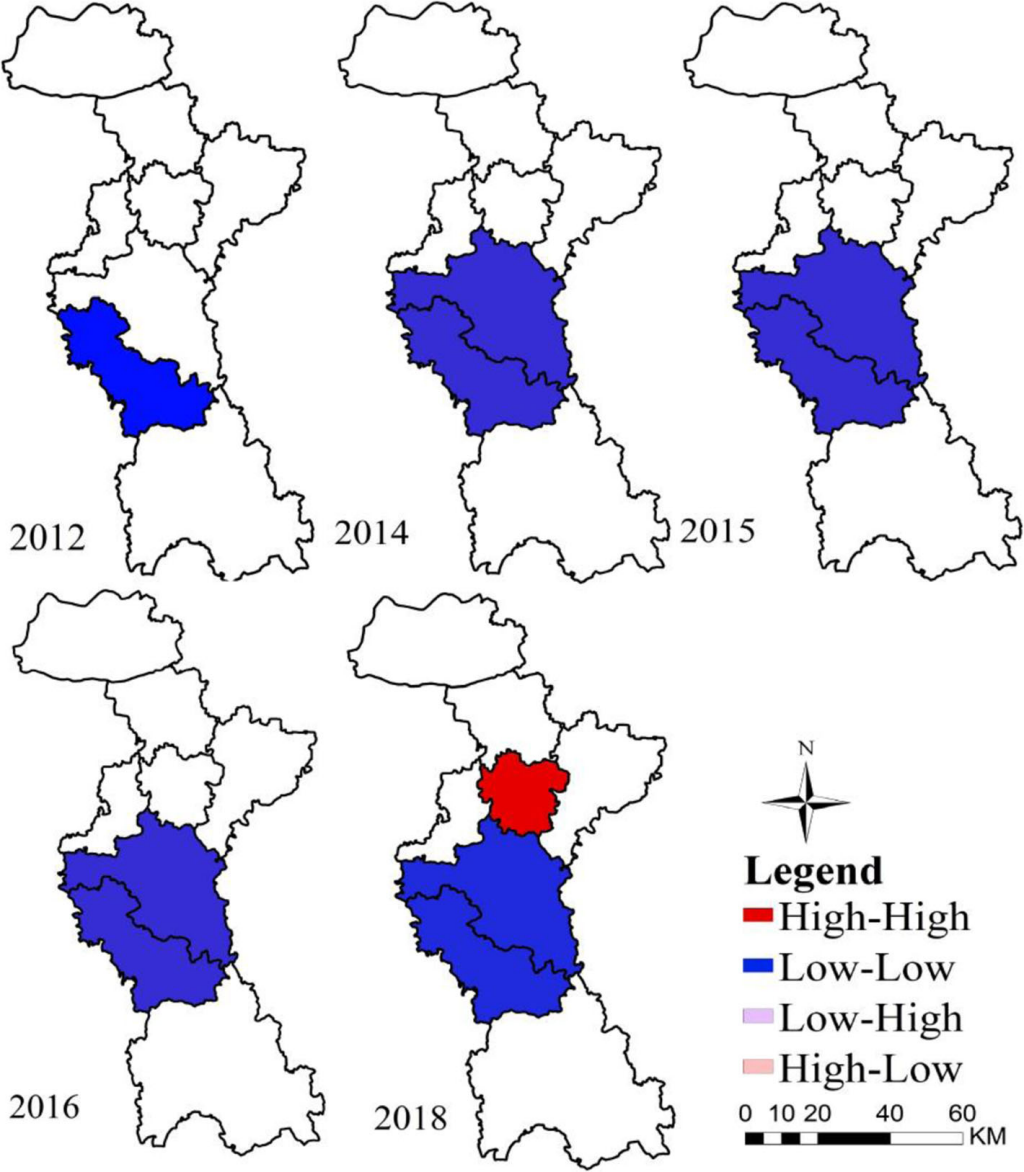
The results of local spatial autocorrelation of HFMD in Zibo city, Shandong Province, 2010–2019 (The author drew this map by ArcGIS10.5 and OpenGeoDa1.2.0 software). There are only two kinds of results, and the same color describes the same kind of cluster areas, red represents hot spots, means the surrounding area and study areas are high incidence areas, blue represents cold spots, and the blank parts were the scanning areas with no statistical significance

The global autocorrelation results were depicted in 2010–2019 (Table 2). Only in 2012, 2014, 2015, 2016 and 2018, the differences were statistically significant (*P* < 0.05), which revealed the HFMD has a significant positive spatial correlation on the county scale in those 5 years in Zibo city, and the distribution of cases was not random in these years, so local autocorrelation analysis was conducted for these 5 years respectively (Fig. 5). According to the results of local spatial autocorrelation combined with LISA map, there were merely a kind of low-low clustering pattern in 2012, 2014, 2015 and 2016. It clustered in Boshan county in 2012, which showed that Boshan District and surrounding areas were also low incidence areas. During 2014–2016, the low-low areas in Zibo city mainly clustered in Boshan county and Zichuan District. In 2018, with the exception of low-low clusters in Boshan county and Zichuan District, high-high cluster appeared and mainly concentrated in Zhangdian District, indicating that the incidence of HFMD in Zhangdian District and surrounding counties were also relatively high. While low-low areas are similar to the situation in 2014–2016, clustering in Boshan county and Zichuan District.

**Table 2:** The global autocorrelation analysis of HFMD in Zibo, Shandong Province, 2010–2019

Year	Moran’ *I*	E(I)	S	*Z* value	*P* value
2010	−0.1387	−0.1429	0.1974	0.0282	0.467
2011	−0.1086	−0.1429	0.2498	0.1426	0.398
2012	0.3241	−0.1429	0.2280	2.0185	0.026*
2013	0.1333	−0.1429	0.1637	1.6599	0.060
2014	0.3549	−0.1429	0.2442	2.0305	0.031*
2015	0.2998	−0.1429	0.2283	1.9711	0.033*
2016	0.2998	−0.1429	0.2356	1.8678	0.041*
2017	0.1390	−0.1429	0.2165	1.2912	0.108
2018	0.5936	−0.1429	0.2427	3.0064	0.005*
2019	0.0013	−0.1429	0.2307	0.6646	0.260

p* represents the value is statistically significant.

### Spatiotemporal clusters analysis

**Fig. 6:**
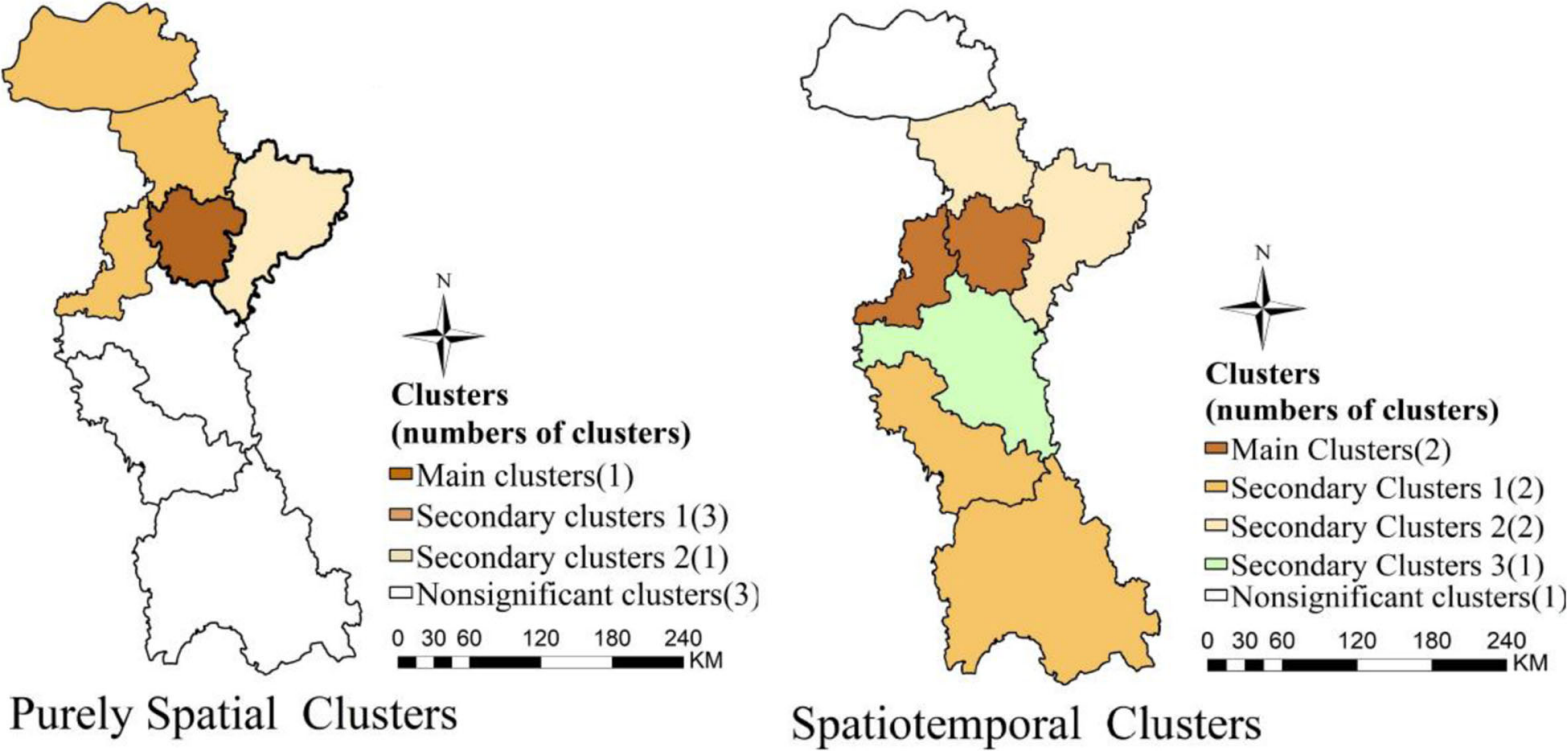
The results of spatiotemporal scan of HFMD from 2010 to 2019 in Zibo city, Shandong Province, in 2010–2019 (The author drew this map by ArcGIS10.5 and SaTscan 9.4 software). The same color describes the same kind of cluster areas, but the blank parts were the scanning areas with no statistical significance

We found that the distribution of HFMD in Zibo city had obvious spatial aggregation characteristic. Purely spatial scanning results revealed that Zhangdian District, located in the north of Zibo city, belongs to main clusters. Secondary cluster1 covered Gaoqing county, Huantai county of the north of Zibo city and Zhoucun county. Secondary cluster 2 included Linzi county of the northeast of Zibo city (Fig. 6). Corresponding to spatial scanning, the main clusters of spatiotemporal scanning located in Zhangdian District (Fig. 6). The difference was that the secondary cluster1 is composed of Boshan District and Yiyuan county in the south of Zibo city. Secondary cluster 2 concentrated in Huantai county and Linzi District and cluster 3 in Zichuan District of the middle of Zibo city, and the aggregation period was from April to September, which was consistent with the seasonal figures of HFMD. In addition, the *RR* value of all clusters were greater than 2, among which that of the main cluster was smallest, indicating that the incidence risk inner the clusters was at least twice as that outside the gathering area (Table 3).

**Table 3:** Spatiotemporal scanning results of HFMD in Zibo city, Shandong Province in 2010–2019

Scanning area	Scanning radius (km)	Date (Year/Month/Day)	Actual cases	Expected cases	*LLR*	*RR*	*P*
Main clusters	33.63	2010/4/12 ~ 2012/9/18	6992	3146.16	1893.06	2.41	< 0.001
1nd secondary	44.17	2010/5/23 ~ 2010/8/3	1524	244.11	1527.20	6.40	< 0.001
2rd secondary	23.89	2010/5/25 ~ 2010/7/27	1187	231.57	993.34	5.22	< 0.001
3nd secondary	0.00	2014/5/31 ~ 2014/8/14	687	160.58	474.85	4.32	< 0.001

*RR* Relative risk; *LLR* Logarithmic Likelihood Ratio

## Discussion

HFMD remains a serious public health problem in Zibo city, and the average annual incidence was up to 129.7/100,000, which threatens the health of children under 5 years old, especially boys. The dominant susceptible population were scattered children, accounting for more than half of cases, similar as other studies [26]. The characteristics mentioned above could mainly related to following explanations: 1) Some children’s antibody obtained from their mothers, have declined year by year from their birth, but their own natural immunity has not yet formed and their resistance is poor. Besides, the bad personal hygiene habits also make them susceptible to infection; 2) Compared to girls, boys have a wider range of activities and are more likely to be in contact with more people, increasing the risk of infection; 3) Scattered children have a wider range of activity scope and poor resistance than the kindergarten and preschool students. Additionally, although the public health agencies have taken measures to protect students in the collective environment from HFMD in terms of regular inspection, case isolation, disinfection of toys and tableware, corresponding unified management and measures for scattered children was lacking. According to data from the 2018 Health Statistics Yearbook (https://www.yearbookchina.com/), Zichuan county and Boshan County are among the counties with the lowest per capita GDP (the third and fourth from the bottom respectively). They are economically underdeveloped and have more migrant workers, so they have few opportunities for population gathering and are not easy to be infected.

Our study shows that, the seasonal feature of HFMD in Zibo city was a unimodal trend, mainly concentrating from April to September, which is consistent with the studies in Beijing city, Qinghai Province and Guangdong Province [24, 27]. Perhaps, it is because the impacts of discrepancy in climatic, geographical factors, the onset of HFMD of different regions present obvious spatial heterogeneity. Different from this study, some studies were a bimodal distribution trend, mainly concentrating in April–July and September–November respectively, as in Hunan Province and Xi’an city [21, 28].

From spatial autocorrelation analysis of the whole populations, we found that Hot spot appears only in Zhangdian District in 2018, indicating that Zhangdian District and surrounding areas were high incidence areas while cold spots clustered in Boshan county and Zichuan District in 2012, 2014, 2015, 2016, and clusters were no statistical significance in other years. Hot spots appeared in Zhangdian District, which proved that HFMD of Zibo city was not randomly distributed. From 2010 to 2019, the total number of HFMD cases showed a decrease trend, most cases concentrated in central and northern Zibo city, showing a distribution that more cases in North than in South of Zibo city. Several possible reasons account for this phenomenon. Firstly, Zhangdian District is the central city of Zibo city, the transportation and communication hub of Middle Shandong with high population density and large number of susceptible people. Its frequent contacts with surrounding cities contributed to greater population mobility and more susceptible population. Secondly, data from Zibo Statistics Bureau (http://tj.zibo.gov.cn/module) showed that Zhangdian District is the county with the highest GDP per capita, in addition, more large general hospitals located in Zhangdian District, especially Zibo Women & Children Hospital. For a better medical conditions, parents in nearby counties will bring their children to Zhangdian District, which increases the chance of contacting with ill children and risk of infected. On the other hand, parents of children in Zhangdian District are more primitive in seeking medical care, and ability of the diagnosis and report cases of medical institutions is stronger, which means more diagnosed cases. From the perspective of housing situation, education resources in the central urban area are relatively excellent, and many households in surrounding counties have bought houses and settled down in Zhangdian District, which leads to increase of children and students. Above reasons may contributed to Zhangdian District becoming the major cluster area in Zibo city. Although Zichuan District and Boshan County are close to Zhangdian District and Zichuan District has large population, the two counties cover a large area, besides the population density is very small, which is not easy to cause crowd gathering, the probability of infection is much smaller, moreover, there is no large general hospital in the two counties, so the medical level is lower than that in Zhangdian District hospital, which were the probable explanation of cold spots clustering and less cases. The spatiotemporal features of an infectious disease are usually driven by certain determinants that can provide invaluable information for exploring the risk factors of the disease and contribute to developing effective measures to control and prevent its transmission.

This study has some limitations. Firstly, Moran’s I used in this study is classic approach to investigate the spatial autocorrelation based on lattice system, however, it is an unadjusted measurement, we did not take the effects of influences factors as mediator on spatial heterogeneity into account, such as per capita GDP, daily mean temperature and relative humidity, etc. If we conducted stratified analysis to adjust for the effects of age and sex, which could generate more 0 values. According to previous published studies, continuous variables cannot be adjusted by hierarchical analysis, but spatial mixed effect models can address the effects of continuous variables on spatial distribution, which was made, such as Bayesian nonparametric approach [29–31], which was taken into account. Secondly, many studies have shown that HFMD could be affected by meteorological factors, social economic conditions and local geography [12, 32, 33], there factors were not mentioned in our study, and will be taken into consideration in the following research. In addition, due to HFMD is self-limited disease, some patients may not have been to the hospital, so cases tracking cannot be done systematically, and the number of patients may be underestimated.

## Conclusions

HFMD mainly threatens children under 5 years old, especially boys, which revealed that the focus of the prevention and control of HFMD in Zibo city should on these populations. The incidence peak is mainly from April to September and spatially clusters concentrated in the central and northern Zibo city. The hot spot was clustered in Zhangdian District in 2018. It is suggested that more manpower and material resources were allotted to prevent and control the high incidence areas of HFMD more effectively, especially in Zhangdian district, the early identification and prevention of high incidence area will improve the efficiency of control and management of HFMD in Zibo city.

## Data Availability

The data that support the findings of this study are available from Zibo CDC but restrictions apply to the availability of these data, which were used under license for the current study, and so are not publicly available. Data are however available from the authors upon reasonable request and with permission of Zibo CDC staff (E-mail: lucywl120@sina.com).

## Abbreviations

HFMD: Hand, foot and mouth disease
RR: Relative risk
LLR: Logarithmic likelihood ratio
GDP: Gross national product
H-H mode: High-high mode
H-L mode: High-low mode
L-L mode: Low-low mode
L-H mode: Low-high mode
